# Left Atrial Strain as a Predictor of Early Anthracycline-Induced Chemotherapy-Related Cardiac Dysfunction: A Pilot Systematic Review and Meta-Analysis

**DOI:** 10.3390/jcm13133904

**Published:** 2024-07-03

**Authors:** Aman Goyal, Haleema Qayyum Abbasi, Shreyas Yakkali, Abdul Moiz Khan, Muhammad Daoud Tariq, Amir Humza Sohail, Rozi Khan

**Affiliations:** 1 Seth G.S. Medical College and KEM Hospital, Mumbai 400012, India; 2 Ayub Medical College, Abbottabad 22020, Pakistan; 3 Jacobi Medical Center, Bronx, NY 10461, USA; 4 Foundation University Medical College, Punjab 44000, Pakistan; 5 University of New Mexico, Albuquerque, NM 87131, USA; 6 Medical University of South Carolina Florence Medical Center, Florence, SC 29505, USA

**Keywords:** chemotherapy-related cardiac dysfunction, anthracyclines, left atrial strain, left ventricular global longitudinal strain, speckle-tracking echocardiography, meta-analysis

## Abstract

**Background**: Chemotherapy-related cardiac dysfunction (CTRCD) significantly affects patients undergoing anthracycline (AC) therapy, with a prevalence ranging from 2% to 20%. Reduced left ventricular ejection fraction (LVEF) and left ventricular global longitudinal strain (LV GLS) are prognostic parameters for CTRCD detection. Our study aimed to investigate the role of emerging parameters such as left atrial strain (LAS). **Methods**: We searched multiple databases for studies comparing LAS changes post-AC versus pre-AC therapy in patients with cancer. Primary outcomes included left atrial reservoir strain (LASr), left atrial conduit strain (LAScd), and left atrial contractile strain (LASct). RevMan (v5.4) was used to pool the standardized mean difference (SMD) under a random effects model, with *p* < 0.05 as the threshold for statistical significance. **Results**: In an analysis of 297 patients across five studies, AC therapy significantly lowered LASr (SMD = −0.34, 95% CI:−0.55, −0.14, I^2^ = 0%, *p* = 0.0009) and LAScd (SMD = −0.41, 95% CI: −0.59, −0.23, I^2^ = 0%, *p* < 0.00001) levels. Conversely, LASct demonstrated no significant change (SMD = 0.01, 95% CI: −0.21, 0.23, I^2^ = 9%, *p* = 0.95). AC therapy also significantly reduced LV GLS (SMD = −0.31, 95% CI: −0.51, −0.11, I^2^ = 0%, *p* = 0.003). While not statistically significant, LVEF decreased (SMD = −0.20, 95% CI: −0.42, 0.03, I^2^ = 0%, *p* = 0.09), and left atrial volume index trended higher (SMD = 0.07, 95% CI: −0.14, 0.27, I^2^ = 0%, *p* = 0.52) after AC therapy. **Conclusions**: AC treatment led to reduced LAS and LV GLS values, indicating its potential as an early CTRCD indicator. Larger trials are required to fully explore their clinical significance.

## 1. Introduction

Over the past few decades, there has been notable advancement in cancer diagnostic techniques and therapeutic interventions, resulting in improved survival rates for patients across various age groups [1]. Nevertheless, it is imperative to acknowledge that both conventional and contemporary antineoplastic treatments have the potential to induce cardiotoxicity, presenting as both short- and long-term complications [1]. Anthracycline (AC) treatment continues to be fundamental in the management of various cancers, such as lymphoma, sarcoma, breast cancer, and pediatric leukemia [1]. However, while it improves long-term cancer survival rates, it also poses notable risks for cardiovascular issues [2]. Despite adherence to dosage limitations, research indicates that anthracycline-induced cardiac dysfunction (ACD) manifests as heart failure in approximately 6% of cases and as subclinical cardiac dysfunction in up to 18% [3].

ACs are the initial choice of medication for lymphoma chemotherapy. They primarily function by blocking DNA and RNA production in cells and inhibiting the activity of topoisomerase II [4]. The incidence of heart-related complications among cancer patients has increased, particularly due to medications such as AC, which have been associated with unforeseen cardiovascular issues such as heart failure [5]. Current guidelines based on the recommendations of the American Society of Echocardiography (ASE) and the European Association of Cardiovascular Imaging (EACVI) describe chemotherapy-related cardiac dysfunction (CTRCD) as a decline in left ventricular ejection fraction (LVEF) of >10% without symptoms or an LVEF below the normal range (LVEF < 53%) following cancer treatment [6]. Numerous studies have emphasized the importance of the prompt recognition of early AC-induced cardiomyopathy. This recognition can potentially aid in restoring left ventricular (LV) function, either by stopping harmful chemotherapy or by initiating cardioprotective measures [7,8]. However, a decline in LVEF represents a late-stage manifestation within the context of cardiotoxicity [9]. In contrast to the traditional use of echocardiographic measurements such as LVEF as a diagnostic criterion [8], recent studies have highlighted speckle-tracking echocardiography (STE) employing myocardial deformation analysis, such as LV global peak systolic longitudinal strain (GLS), as a viable, objective, and better approach for identifying subtle yet clinically significant LV systolic dysfunction [10].

Recently, left atrial (LA) strain has emerged as an efficient indicator of ‘subclinical’ or early diastolic dysfunction [11]. Studies have shown it to be a more accurate marker of LV filling pressures than conventional Doppler-derived diastolic parameters [12]. LA strain serves as a junction between the pulmonary and systemic circulation, regulating LV loading, and thus is hypothesized to provide a more accurate representation of LV diastolic function [11]. According to experts, ventricular diastolic dysfunction often precedes the onset of systolic impairment [6]. While LVGLS using 2D STE is considered sensitive for LV dysfunction in cancer patients, the role of LA strain (LAS) remains underexplored; therefore, our pilot study aimed to assess the changes in LAS as an early predictor of AC-induced CTRCD in cancer patients.

## 2. Materials and Methodology

This meta-analysis was conducted following the guidelines outlined by the Cochrane Collaboration (UK) [13] and adhered to the reporting standards set by the Preferred Reporting Items for Systematic Review and Meta-Analysis Statement (PRISMA 2020) [14]. The study protocol was registered in the PROSPERO International Prospective Register of Systematic Reviews with an identification number (CRD42024540918).

### 2.1. Data Sources and Search Strategy

We conducted a comprehensive electronic search across multiple databases, including MEDLINE via PubMed, EMBASE, clinicaltrial.gov, Google Scholar, SCOPUS, and other relevant sources of the literature, from their inception to April 2024, to retrieve studies reporting changes in LAS as an early predictor of AC-induced CTRCD in cancer patients. Predefined Medical Subject Headings (MeSH) terms combined with the Boolean operators ‘AND’ and ‘OR’ were utilized. The search strategy used the following terms: ‘Left atrial strain’, ‘LA strain’, ‘Left atrial longitudinal strain’, ‘Cardiac Toxicity’, ‘chemotherapy-related cardiac dysfunction’, ‘chemotherapy-induced cardiotoxicity’, ‘Anthracyclines’ and ‘ANT’. There were no language or timeframe restrictions during the search. Additionally, we manually searched the reference lists to ensure completeness, and the detailed search strategy can be found in the Supplementary Materials file.

### 2.2. Study Assessment

Two investigators (H.Q.A. and A.M.K.) independently evaluated the titles and abstracts of potential studies to determine their eligibility, excluding duplicates or those not meeting the predefined inclusion criteria. Subsequently, the full text of the selected articles was carefully examined to exclude studies that did not meet the predetermined eligibility criteria. Any disagreements were resolved through discussion led by a third reviewer to achieve a consensus (A.G.). There were no limitations to the sample size or follow-up duration.

### 2.3. Eligibility Criteria

The inclusion criteria were based on the PICOS format for systematic reviews and meta-analyses, with the population (P) being cancer patients treated with AC, the exposure (E) being post-AC therapy, the control (C) being pre-AC therapy, and the outcomes (O) being various parameters such as LA reservoir strain (LASr), LA conduit strain (LAScd), LA contractile strain (LASct), LVEF, LVGLS, and LA volume indexed (mL/m^2^) (LAVI).

The exclusion criteria were as follows: (1) studies that fit into any of the following categories: case reports and series, literature reviews, systematic reviews, meta-analyses, or letters to editors; further exclusion criteria included (2) studies in which patients did not receive ACs at any point during their treatment; (3) lack of echocardiograms before or after AC exposure; (4) patients with prior cardiac conditions such as myocardial infarction, myocarditis, severe valvular disease, atrial fibrillation, or atrial flutter; (5) echocardiography lacking an apical 4-chamber view or poor image quality due to extreme obesity or severe pneumonia; (6) studies using animal models; and (7) studies lacking adequate clinical data pertinent to the outcomes being studied.

### 2.4. Data Extraction and Quality Assessment

Two researchers (H.Q.A. and A.M.K.) extracted data from the included studies using a pre-established Microsoft Excel spreadsheet. If there were discrepancies, an impartial researcher (A.G) reviewed the data. The extracted data included the first author’s name, publication year, country of origin, study design, sample size (both intervention and control groups), type of cancer, cumulative AC dose, and reported outcomes. For the quality assessment of the included studies, two researchers (H.Q.A and A.M.K) independently used the Newcastle–Ottawa Quality Assessment Tool [15]. Bias was assessed as low (7–10 points), moderate (4–6 points), and high (0–3 points).

### 2.5. Endpoint Definition

The primary endpoints were the changes in (1) LASr (%), (2) LAScd (%), and (3) LASct (%). The secondary endpoints included (4) LEVF, (5) LV GLS, and LAVI (6).

### 2.6. Data Synthesis

The collected data were pooled and analyzed using Review Manager Version 5.4 (Cochrane Collaboration, UK) by two authors (A.G. and H.Q.A.). The standardized mean difference (SMD) between the follow-up and baseline values was used for continuous outcomes. Generic invariance random-effects models were employed for continuous outcomes, and a *p*-value of less than 0.05 was considered statistically significant. Statistical heterogeneity was assessed using Higgins I^2^ statistics, where <25%, 25–75%, and >75% indicated low, moderate, and high heterogeneity, respectively. Publication bias was assessed through visual inspection of funnel plots.

## 3. Results

### 3.1. Literature Search Results

**Figure 1:**
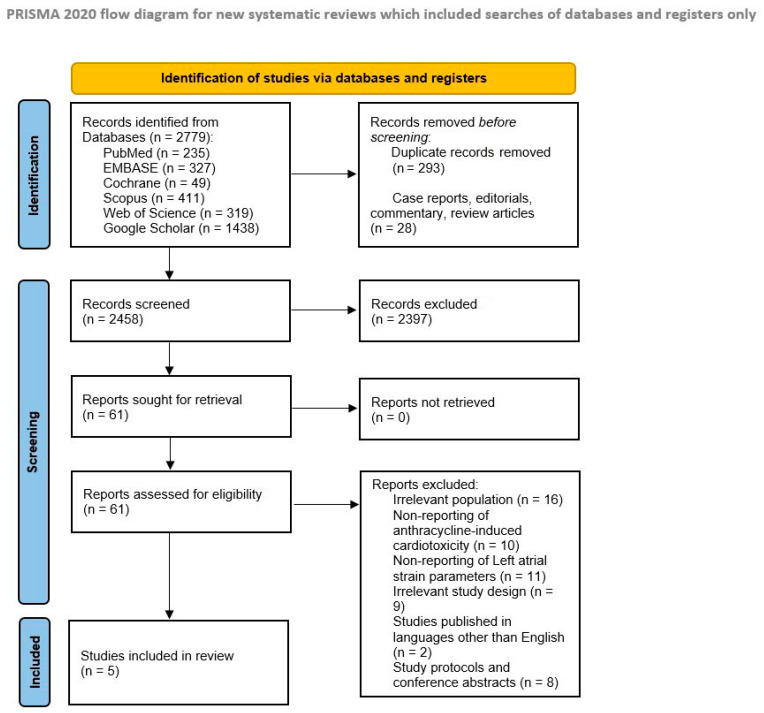
Preferred Reporting Items for Systematic Reviews and Meta-Analyses (PRISMA) Flow Diagram (2020) for Systematic Reviews and Meta-Analyses.

Using our search strategy, 2779 studies were identified from six electronic databases. After eliminating duplicates, correspondence, case reports, and review articles, 321 articles were excluded. Following the initial screening based on the title and abstract, 2397 studies were excluded as they did not meet the specified criteria. This left 61 articles for full-text reviews. Among these, 56 studies were further excluded for various reasons, including irrelevant populations, lack of pertinent outcomes, unsuitable study designs, study protocols, conference abstracts, and non-English language full texts. As a result, only five studies that fulfilled the eligibility criteria were included in our meta-analysis [16,17,18,19,20]. A detailed summary of the literature search and study selection process is shown in Figure 1.

### 3.2. Study Characteristics and Risk of Bias Assessment

Our review encompassed studies conducted from 2018 to 2023. Among the included studies, four employed a prospective cohort design [16,17,18,19], whereas one utilized a retrospective design [20]. These studies were conducted in China [16], Australia [17], Israel [18], Canada [19], and the United States [20]. Three studies included patients with breast cancer [17,18,19], one included patients with lymphoma [16], and one focused on various malignancies [20], including osteosarcoma, Ewing’s sarcoma, and leukemia. The combined cohort consisted of 297 cancer patients undergoing AC therapy. The detailed characteristics of all included studies and baseline characteristics of the patients are presented in Table 1 and Table 2, respectively.

Each study underwent rigorous quality assessment, achieving a score of 7 or higher on the Newcastle–Ottawa Scale (Supplementary Materials Table S1).

### 3.3. Outcomes

Our study reported six outcomes.

#### 3.3.1. Primary Outcomes

**Figure 2:**
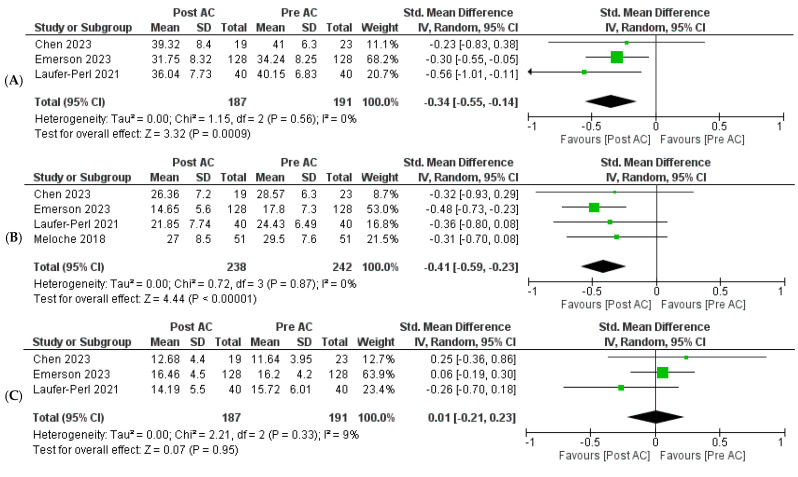

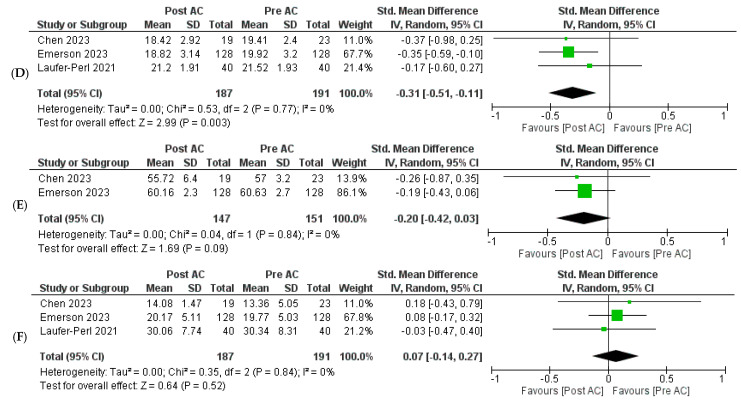
Forest plots were employed for the pooled analysis of data. Risk Ratio (RR) was used for binary variables, with statistical significance set at a 95% confidence interval (CI). The weight of each included study is represented by the size of the green square adjacent to the study, and the black diamond illustrates the results of the pooled analysis of all studies. The width of the black diamond represents the pooled confidence interval. Forest plots were generated for various outcomes, including LASr (**A**), LAScd (**B**), LASct (**C**), LV GLS (**D**), LVEF (**E**), and LAVI (**F**) [16,17,18,19].

Three of the five included studies provided data on LASr in patients undergoing AC therapy [16,17,18]. Our meta-analysis demonstrated a notable and statistically significant reduction in LASr following AC therapy [SMD = −0.34, 95% CI: −0.55 to −0.14, *p* = 0.0009]. No heterogeneity was found among the studies (I^2^ = 0%, *p* = 0.56) (Figure 2A). Four of the five included studies reported the outcome of LAScd [16,17,18,19], and our pooled analysis indicated that AC therapy was associated with a significant reduction in LAScd [SMD = −0.41, 95% CI: −0.59 to −0.23, *p* < 0.00001]. No heterogeneity was found among the studies (I^2^ = 0%, *p* = 0.87) (Figure 2B). Three of the five included studies reported LASct as an outcome [16,17,18]. The meta-analysis indicated no statistically significant change in LASct following AC therapy [SMD = 0.01, 95% CI: −0.21 to 0.23, *p* = 0.95]. Mild heterogeneity was reported among the studies (I^2^ = 9%, *p* = 0.33) (Figure 2C).

#### 3.3.2. Secondary Outcomes

Three of the five included studies investigated the effect of AC therapy on LV GLS [16,17,18]. Pooled analysis revealed that AC therapy significantly reduced LV GLS values [SMD = −0.31, 95% CI: −0.51 to −0.11, *p* = 0.003]. No heterogeneity was found among the studies (I^2^ = 0%, *p* = 0.77) (Figure 2D). Two of the five included studies reported LVEF outcomes [16,17]. Our meta-analysis did not find a statistically significant effect on LVEF following AC therapy [SMD = −0.20, 95% CI: −0.42 to 0.03, *p* = 0.09]. No heterogeneity was found among the studies (I^2^ = 0%, *p* = 0.84) (Figure 2E). Three of the five included studies reported LAVI outcomes in patients undergoing AC therapy [16,17,18]. Our meta-analysis indicated no statistically significant change in LAVI following AC therapy [SMD = 0.07, 95% CI: −0.14 to 0.27, *p* = 0.52]. No heterogeneity was found among the studies (I^2^ = 0%, *p* = 0.84) (Figure 2F).

### 3.4. Publication Bias

The risk of publication bias was assessed using visual inspection of funnel plots (Supplementary Materials Figure S1). All outcomes indicated a low risk of bias.

## 4. Discussion

ACs, such as doxorubicin and daunorubicin, are potent chemotherapeutic medicines used to treat a variety of cancers, including lymphoma, breast cancer, and pediatric leukemia [1]. However, their use is limited due to the risk of dose-dependent cardiotoxicity, which can result in both acute and chronic cardiac dysfunction [21]. The precise mechanism of ACD is unknown; however, it is thought to involve a number of mechanisms, including oxidative stress, mitochondrial malfunction, and cardiomyocyte death [21]. Recent research challenges the traditional classification of AC toxicity, suggesting that it is a continuous process rather than a distinct stage of acute, early, or late chronic cardiotoxicity [21]. It is now understood that AC-induced cardiotoxicity begins at the myocardial cell level and progresses to overt heart failure [21]. This implies that we may observe different stages of the same phenomenon rather than three separate conditions [21]. Practically speaking, ACD is now assumed to occur at the moment of initial exposure, which is supported by the discovery of troponin release upon AC delivery [21]. Clinical symptoms may not manifest until years after the initial injury, making diagnosis delayed and sometimes causing it to take years. Considering the decline in LVEF, diagnosis may take months [22,23]. Therefore, recent studies have investigated STE to detect strain patterns and to identify ACD earlier than when using other parameters. In echocardiography, ‘strain’ is a measure of myocardial deformation or the change in the length of the myocardium compared to its original length [24,25]. Various types of strain measures are used in echocardiography, the most popular being longitudinal, radial, and circumferential strains. STE enables the quantitative measurement of these strain patterns [24]. In this pilot systematic review and meta-analysis, we sought to determine the usefulness of LA strain as a predictor of early AC-induced CTRCD in cancer patients.

The LA has three main functions [25]. The first is the reservoir function when it receives oxygenated blood from the pulmonary veins during ventricular systole [25]. Second, the conduit function passively transfers blood from the left atrium to the left ventricle during early ventricular diastole [25]. Lastly, the contractile function contracts actively during late ventricular diastole to aid in left ventricular filling [25].

This study’s first primary outcome was to look at LASr, a positive atrial strain that peaks in systole near the conclusion of LA filling before the mitral valve opens [25]. The first study was conducted by Chen et al., who aimed to assess LV strain patterns, particularly GLS, in 23 children diagnosed with lymphoma, compared to 23 healthy children of similar age recruited from pediatric clinics [16]. Among these children, 20 were male and 3 were female, with a mean age of 9.34  ±  3.59 years (range, 5–15 years). The study concluded that LA strain could serve as an early monitoring tool for cardiotoxicity in children with lymphoma following chemotherapy [16]. The second study was by Emerson et al., who conducted a prospective cohort study involving 128 HER2-negative breast cancer patients who received AC treatment. The study found that in all patients, LASr and LAScd were significantly lower post-AC treatment and moderately correlated with LV diastolic parameters. The reduction in LA strain post-AC treatment was evident even in patients with preserved LV systolic and diastolic functions. More patients showed alterations in diastolic function (≥15% reduction in LASr from baseline) (32%) than in systolic function (≥15% reduction in LV GLS) (23%). The study concluded that LA strain could serve as a promising marker of early diastolic dysfunction in breast cancer patients receiving AC treatment [17]. The third study was conducted by Laufer-Perl et al., who conducted a prospective study involving 40 patients with breast cancer receiving AC therapy. They evaluated LASr and found that reductions were common and occurred early in the course of the AC therapy. These reductions were significantly correlated with the routine echocardiographic diastolic parameters [18]. These findings were in line with our own analyses. 

The next primary outcome, LAScd, was assessed in all aforementioned studies, as well as by Meloche et al. They conducted a prospective cohort analysis of 51 female patients (mean age 50.5 ± 9.8 years) with HER2+ breast cancer who underwent sequential AC treatment. All subjects’ hearts were anatomically sound, with no history of atrial fibrillation. The study found that individuals with CTRCD had poorer baseline left atrial reservoir and conduit function than those without CTRCD [19], consistent with our own findings. In our analysis, we also investigated the effects of AC therapy on LASct. Our findings indicated no statistically significant change in LASct after AC therapy. It is important to note that there is a possibility that the lack of statistical significance could also be due to the outcome being underpowered, given the inclusion of only three studies. In contrast, a study by Inoue et al. comparing LA strain parameters in patients with and without AC-induced cardiotoxicity reported that those who developed cardiotoxicity also had lower LAct and LAsr levels [26]. On the other hand, a study by Giang et al. demonstrated a significant reduction only in LASr levels in patients with AC-induced cardiotoxicity, with no difference observed in either contractile or conduit strain parameters [27]. On the other hand, the fifth study included in our analysis was conducted by Patel et al. They evaluated fifty-five children (mean age 13 [SD 5] years) treated with AC. Their findings revealed that after AC exposure, the LA ejection fraction, LV GLS, and peak GLS rate were reduced [20].

In this study, we investigated several secondary outcomes to further understand the impact of AC therapy on cardiac function. Specifically, we analyzed the effects of AC therapy on LV GLS, LVEF, and LAVI. Our meta-analysis revealed a significant reduction in LV GLS following AC therapy, which is in line with previous studies reporting LVGLS as a viable early prognostic indicator of CTRCD [10]. However, we did not find a statistically significant effect of AC therapy on LVEF, and it is difficult to ascertain if the insignificance was due to the fact that significant CTRCD did not occur at the time of follow-up in the studies included or if the outcome was underpowered to detect adequate statistical significance. In a study by Anqi et al., the authors reported a reduction in both LVEF (consistent with our findings) and LV GLS (in contrast to our findings) with an increasing cumulative dose of AC therapy in patients with breast cancer [28]. The outcome of LAVI trended higher but was not statistically significant, which is in line with increased LAVI being an indicator of LA atriopathy [10].

Diastolic dysfunction and an increase in LV filling pressure occur in the early stages of several types of heart failure. A thorough examination of diastolic function using traditional criteria is also indicated, and prior investigations have revealed early alterations in diastolic functional parameters [29,30]. Until now, none of the conventional parameters of diastolic function have been conclusively shown to correlate well with subsequent LV dysfunction. Our study is the first meta-analysis to highlight the changes observed in LASr, LAScd, and LASct in patients receiving AC therapy to help predict AC-induced CTRCD. Similar to LV GLS, LASr, LAScd, and LASct provide a comprehensive analysis of the global myocardial function of the LA. Importantly, these LA strain patterns may reflect impairment of the mechanical function of the LA muscle before general structural changes occur, such as LA enlargement due to chronic elevation of LV filling pressure. This suggests that LASr, LAScd, and LASct may serve as early markers for the detection of cardiac dysfunction in patients undergoing AC cancer therapy. Our study suggests that LA strain patterns help with ‘more subclinical and earlier’ LV dysfunction and subsequent LA dysfunction, or they can show myocardial dysfunction of the LA itself in cancer patients receiving AC chemotherapy to help in the early prediction of CTRCD before frank changes in LVEF or clinical symptoms appear. One possible explanation for why diastolic parameters have not consistently shown serial changes in the cardio-oncology setting to date is that many patients with cancer experience reduced oral intake and frequently require fluid therapy, particularly during chemotherapy. 

Furthermore, the presence of a ’gray zone’ in LV filling pressure could obscure subtle changes in diastolic function in patients with cancer. These subtle changes may be revealed using the LA strain patterns. Additionally, volumetric analyses of the LA can be easily confounded by measurement errors when performing conventional parameters of diastolic function. The conventional parameters, for example, LAVI, might not adequately reflect subclinical changes in diastolic function and LA function in patients undergoing chemotherapy. Therefore, LA strain patterns, being the most sensitive parameters, provide better insight into early changes in cardiac function in this patient population.

## 5. Limitations

This study should be interpreted in the context of its design, and several limitations should be acknowledged. First, it is worth noting that the majority of the literature on this topic is observational, with no randomized controlled trials identified. This highlights the difficulties in conducting controlled experiments in this area, likely due to ethical concerns. Additionally, observational studies have inherent limitations, including unmeasured confounders and between-study heterogeneity, although our study found minimal to no heterogeneity in our results.

Secondly, although most of our outcomes reported low heterogeneity between studies in our analyses, some heterogeneity still exists, which can be attributed to varying patient pathologies and age groups in the included studies. This warrants further research to be conducted on specific pathologies and age groups to generate more robust data with better generalizability. Furthermore, variations in the characteristics of the populations studied, such as demographic differences, comorbidities, and baseline health conditions, limit the generalizability of our results. This is particularly important as some observational studies were conducted in children, while others were in adults. Variability is also introduced by differences in study designs, such as retrospective versus prospective approaches, variations in cohort sizes, and differences in data collection methods.

Lastly, it is important to note that the cutoff for strain patterns used in different studies varied, which could affect the comparability of results across studies. Therefore, future research should aim for more standardized methodologies, including set definitions for LA strain reduction percentages within more homogeneous populations. Planning for randomized controlled trials in this aspect would further enhance the strength and generalizability of the findings.

## 6. Conclusions

In conclusion, our pilot systematic review and meta-analysis provide valuable insights into the role of LA strain as a predictor of early AC-induced CTRCD. Our findings suggest that AC therapy is associated with a significant reduction in LASr and LAScd, indicating early impairment of LA function. Importantly, these reductions in LA strain parameters seem to occur before significant changes in LVEF and clinical symptoms appear, highlighting the potential of LA strain as an early marker for the detection of CTRCD. Further research is warranted to validate our findings, and future prospective studies should compare LA strain and LV GLS to determine which is a better prognostic marker and a superior early predictor of CTRCD in the cardio-oncology setting.

## Figures and Tables

**Table 1:** Study characteristics of the included studies.

Study ID	Country	Study Design	Type of Cancer n (%)	Type of Anthracycline n (%)			
				Doxorubicin	Daunorubicin	Epirubicin	Other
Chen et al., 2023 [16]	China	Prospective cohort study	Lymphoma = 23 (100)	23 (100)	0	0	0
Emerson et al., 2023 [17]	Australia	Prospective cohort study	HER2-negative breast cancer = 128 (100)	NR	0	NR	0
Laufer-Perl et al., 2021 [18]	Israel	Prospective cohort study	Hormone receptor-positive breast cancer = 21 (52.5) HER2-positive breast cancer = 11 (27.5) Triple-negative breast cancer = 8 (20)	40 (100)	0	0	0
Meloche et al., 2018 [19]	Canada	Prospective cohort study	HER2-positive breast cancer = 51 (100)	0	0	51 (100)	0
Patel et al., 2018 [20]	United States	Retrospective cohort study	Osteosarcoma = 19 (36) Ewing sarcoma = 11 (21) Leukemia = 8 (15) Other = 15 (28)	40 (78)	7 (14)	NR	4 (8)

Legend: NR = not reported.

**Table 2:** Baseline characteristics of the patients.

Study ID	Total No. of Participants	Age in Years (Mean SD)	Female n (%)	Weight (kg)	BMI (Mean, SD)	Hypertension n (%)	Diabetes Mellitus n (%)	Hyperlipidemia n (%)	Smoking n (%)	Cumulative Anthracycline Dosage (mg/m^2^) (Mean ± SD)	Time Elapsed between Baseline and Post-AC Therapy Echocardiograms (Months)
Chen et al., 2023 [16]	23	9.34 ± 3.59	3 (13)	29.28 ± 14.22	NR	NR	NR	NR	NR	50	NR
Emerson et al., 2023 [17]	128	54.7 ± 9.6	NR	74.23 ± 14.92	29.05 ± 6.52	39 (30)	9 (7)	27 (21)	38 (30)	195.6 ± 73.5	3
Laufer-Perl et al., 2021 [18]	40	51 ± 12	40 (100)	NR	27 ± 6	12 (30)	3 (7.5)	8 (20)	5 (12.5)	237 ± 13.24	3.18 ± 0.62
Meloche et al., 2018 [19]	51	50.5 ± 9.8	51 (100)	NR	NR	NR	NR	NR	NR	300	NR
Patel et al., 2018 [20]	55	13 ± 5	12 (22)	NR	NR	NR	NR	NR	NR	308.6 ± 152	10.3 ± 8.7

Legend: SD = standard deviation; BMI = body mass index; NR = not reported; AC = Anthracycline.

## Data Availability

The original contributions presented in the study are included in the article/Supplementary Material, further inquiries can be directed to the corresponding author.

## Supplementary Materials

The following supporting information can be downloaded at: https://www.mdpi.com/article/10.3390/jcm13133904/s1, Table S1: Risk of bias summary of included observational studies using Newcastle-Ottawa Scale; Figure S1: Funnel plots to assess for publication bias of included studies.

## Abbreviations

Anthracycline	AC
Anthracycline-induced cardiac dysfunction	ACD
American Society of Echocardiography	ASE
Chemotherapy-related cardiac dysfunction	CTRCD
European Association of Cardiovascular Imaging	EACVI
Left atrial strain	LAS
Left atrial reservoir strain	LASr
Left atrial conduit strain	LAScd
Left atrial contractile strain	LASct
Left atrial volume index	LAVI
Left ventricular ejection fraction	LVEF
Left ventricular global longitudinal strain	LV GLS
Standardized mean difference	SMD
Speckle-tracking echocardiography	STE
