# Supplementary material for: Left Atrial Strain as a Predictor of Early Anthracycline-Induced Chemotherapy-Related Cardiac Dysfunction: A Pilot Systematic Review and Meta-Analysis
Source: J Clin Med. 2024 Jul 3;13(13):3904. doi: 10.3390/jcm13133904 (PMC11242155; doi:10.3390/jcm13133904)
Supplement: Supplementary file 1 [file jcm-13-03904-s001.zip › jcm-3050705-supplementary.pdf]

SUPPLEMENTARY DATA FILE

Search strategy:

	Search string
#1	("Left atrial strain" OR "LA strain" OR "Left atrial longitudinal strain" OR "peak LA longitudinal strain" OR "PALS" OR "LA GLS" OR "LA radial strain" OR "Left atrial radial strain" OR "Left atrial circumferential strain" OR "LA circumferential strain")
#2	(Cardio toxicities OR "Cardiac Toxicity" OR "Cardiac Toxicities" OR "Cancer therapeutics-related cardiac dysfunction" OR "cardiac dysfunction" OR "chemotherapy-induced cardiotoxicity" OR "chemotherapy-induced cardiac dysfunction")
#3	("Anthracyclines"[Mesh] OR "Anthracycline" OR "ANT" OR "Aclarubicin" OR "Daunorubicin" OR "Carubicin" OR "Doxorubicin" OR "Idarubicin" OR "Nogalamycin" OR "Plicamycin" OR "Pradimicins" OR "Benanomicin" OR "Epirubicin")
#4	#1 AND #2 AND #3

Complete search string

("Left atrial strain" OR "LA strain" OR "Left atrial longitudinal strain" OR "peak LA longitudinal strain" OR "PALS" OR "LA GLS" OR "LA radial strain" OR "Left atrial radial strain" OR "Left atrial circumferential strain" OR "LA circumferential strain") AND (Cardio toxicities OR "Cardiac Toxicity" OR "Cardiac Toxicities" OR "Cancer therapeutics-related cardiac dysfunction" OR "cardiac dysfunction" OR "chemotherapy-induced cardiotoxicity" OR "chemotherapy-induced cardiac dysfunction") AND ("Anthracyclines"[Mesh] OR "Anthracycline" OR "ANT" OR "Aclarubicin" OR "Daunorubicin" OR "Carubicin" OR "Doxorubicin" OR "Idarubicin" OR "Nogalamycin" OR "Plicamycin" OR "Pradimicins" OR "Benanomicin" OR "Epirubicin")

Table S1: Risk of bias summary of included observational studies using Newcastle-Ottawa Scale

	**Selection**				**Comparability**	**Outcome**			
**Study**	**Representative of exposed cohorts**	**Selection of non-exposed cohort**	**Ascertainment of exposure**	**Demonstration that outcome of interest was not present at the start of study**	**Comparability of cohort on the basis of design or analysis**	**Assessment of outcome**	**Was follow-up long enough for the outcomes to occur**	**Adequacy of follow-up of cohorts**	**Total score**
Laufer-Perl et al.	*		*	*	*	*	*	*	7
Emerson et al.	*		*	*	*	*	*	*	7
Chen et al.	*	*	*	*	**	*	*	*	9
Patel et al.	*		*	*	*	*	*	*	7
Meloche et al.	*		*	*	*	*	*	*	7

*Score 7 or higher was considered as a good quality study with low risk of bias

Figure S1: Funnel plots to assess for publication bias of included studies

A) LASr

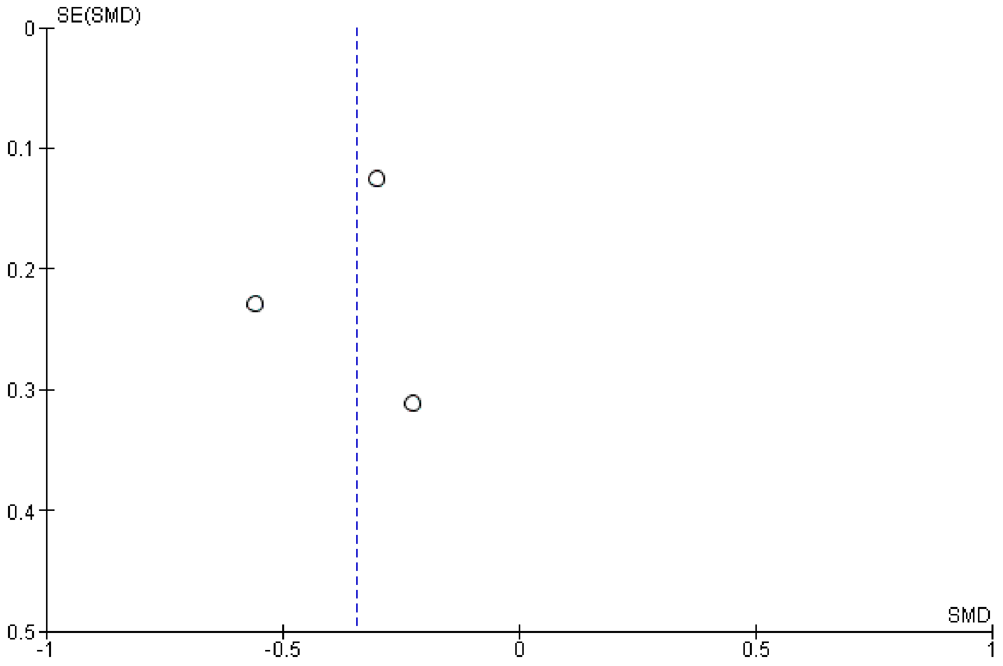

B) LAScd

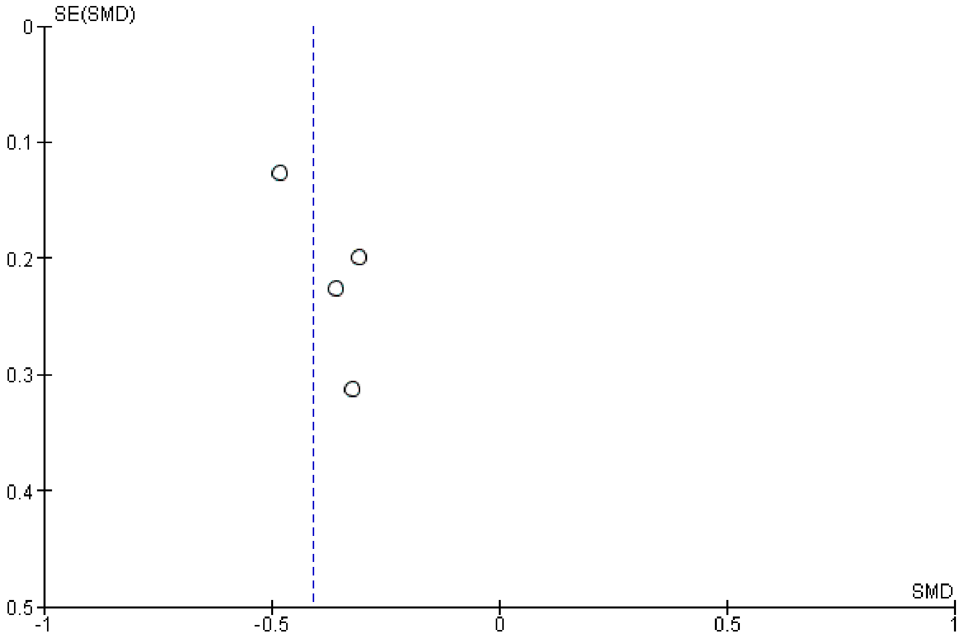

C) LASct

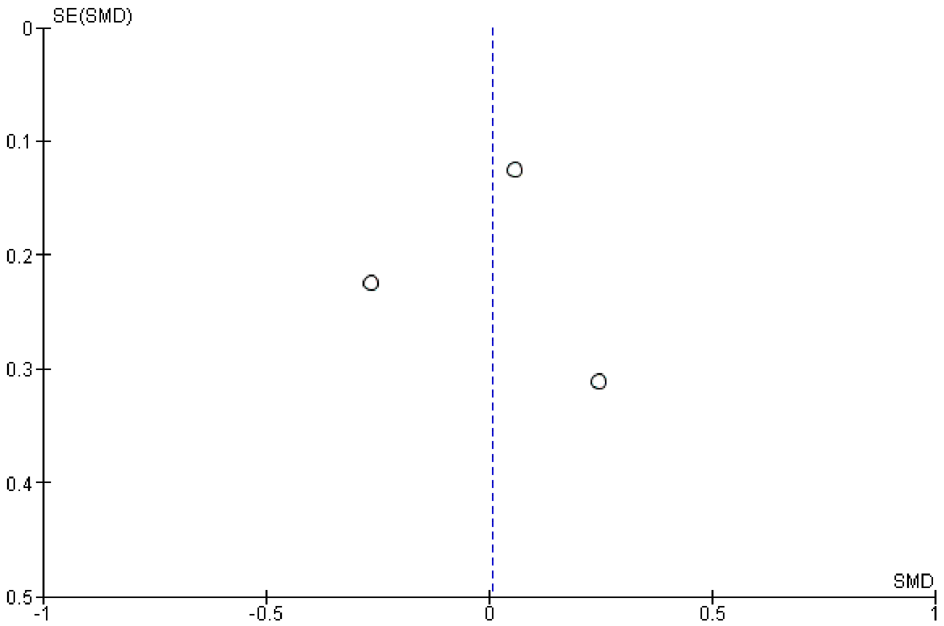

D) LV GLS

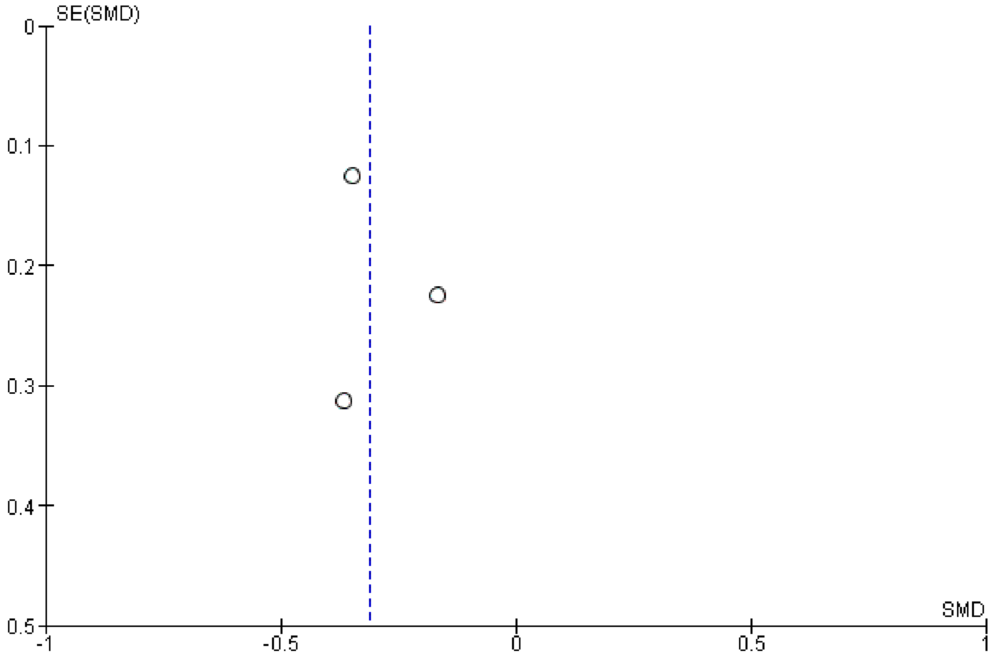

E) LVEF

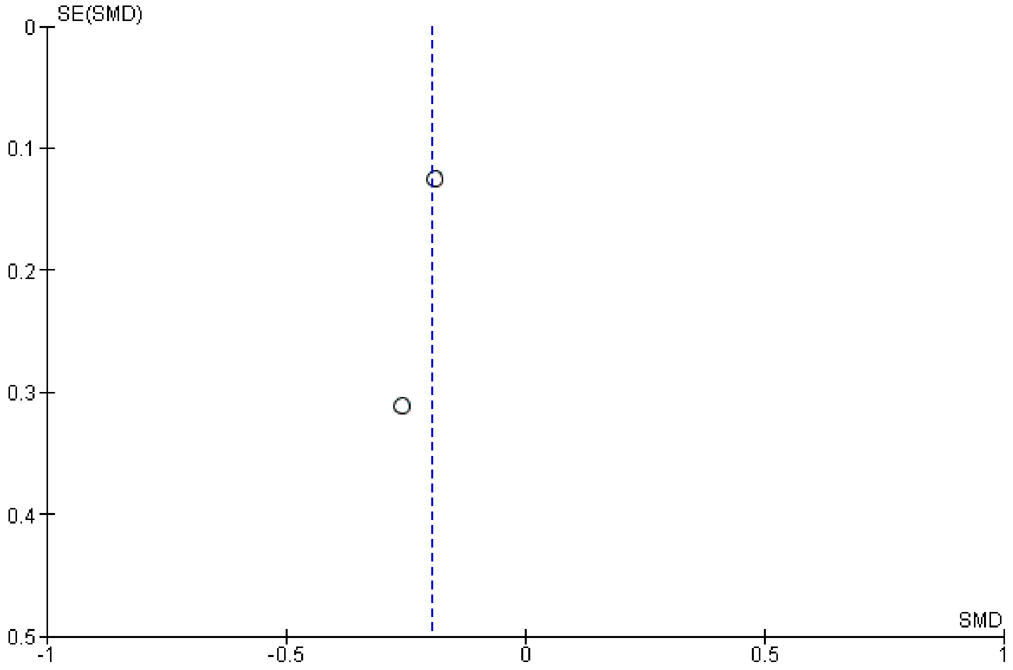
SE(SMD)
0
0.1
0.2
0.3
0.4
0.5
SMD
-1
-0.5
0
0.5
1

F) LAVI

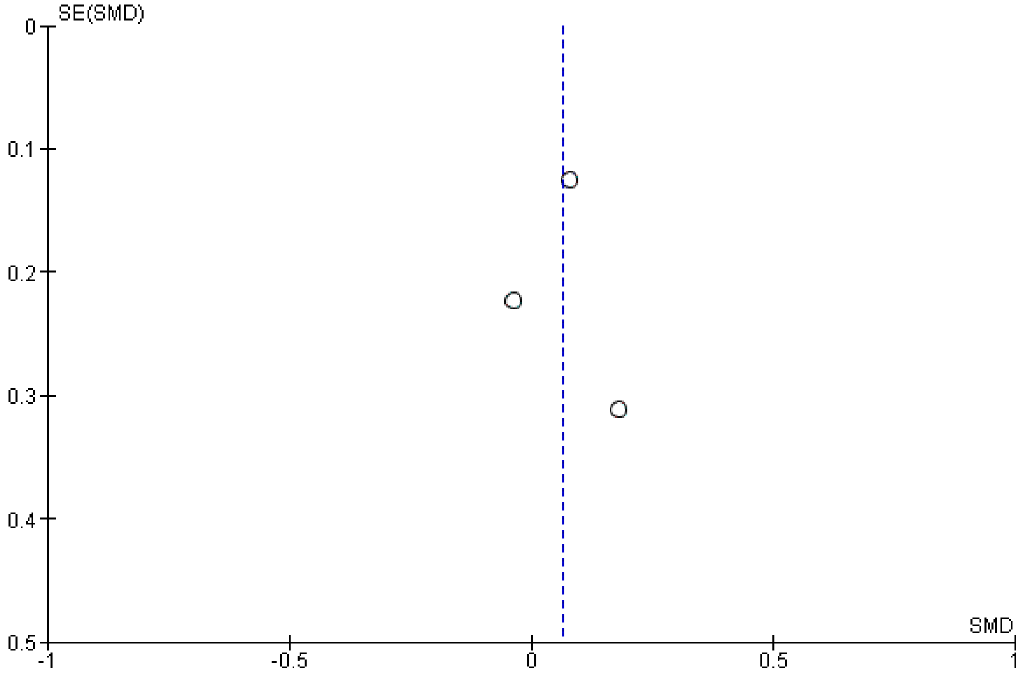
SE(SMD)
0
0.1
0.2
0.3
0.4
0.5
SMD
-1
-0.5
0
0.5
1
